# Current Practices for Evaluating Clinical Supervisors in North American Genetic Counseling Programs

**DOI:** 10.1002/jgc4.70280

**Published:** 2026-08-23

**Authors:** Sydney Schulz, Tasha Wainstein, Susan Randall Armel, Jenna Scott, Tracey Oh

**Affiliations:** ^1^ Department of Medical Genetics, Faculty of Medicine University of British Columbia Vancouver British Columbia Canada; ^2^ Departments of Pediatrics and Medical Genetics, Faculty of Medicine University of British Columbia Vancouver British Columbia Canada; ^3^ BC Children's Hospital Research Institute Vancouver British Columbia Canada; ^4^ Department of Molecular Genetics University of Toronto Toronto Ontario Canada; ^5^ Bhalwani Familial Cancer Clinic Princess Margaret Cancer Centre Toronto Ontario Canada; ^6^ Genetic Counselling Program University of British Columbia Vancouver British Columbia Canada

**Keywords:** clinical supervision, genetic counseling, peer evaluation, peer support, self‐assessment, student evaluation of teaching

## Abstract

Clinical supervision is a cornerstone of genetic counseling education, yet there are no standardized methods for evaluating supervisors across North American programs. The Accreditation Council for Genetic Counseling requires that supervisors be evaluated regularly but provides minimal guidance on implementation. This study explored how genetic counseling programs evaluate their clinical supervisors and examined the perceived effectiveness and limitations of these approaches. 16 out of a possible 64 fieldwork directors (FDs) from accredited genetic counseling programs across the United States and Canada completed semi‐structured interviews. Participants described their evaluation practices and were asked to share their relevant evaluation tools. Using a constructivist paradigm, we analyzed interview data using inductive content analysis followed by interpretive description. All 16 programs used student evaluations; three used self‐assessments; one used peer evaluation; and none used management reviews. The content of the student evaluations as described by FDs addressed aspects of the learning environment including the supervisory relationship, clinical teaching ability, and the clinical site experience. Document analysis indicated broad alignment between the intended purpose of student evaluations and survey content, while also highlighting considerable variability in survey design. Self‐assessment and peer evaluation were rarely implemented but were largely considered positive. Challenges to supervisor evaluation included limited resources, relational dynamics within supervision, and concerns about retaliation. While supervisor evaluation is universally practiced, inconsistent implementation and tool design may limit its value for both students and supervisors. These findings demonstrate the need to clarify the specific purpose of student evaluations in order to develop validated tools and ensure alignment between supervisory practice, training, and goals. Improving supervisor evaluation may enhance both the quality of supervision and the learning environment for students.

## Abstract (highlights)

**What Is Known About This Topic:**

Genetic counseling programs are expected to evaluate their clinical supervisors and provide opportunities to students to give anonymous feedback on their experience of supervision.

**What This Paper Adds to the Topic:**

This paper outlines the common methods used by genetic counseling programs to evaluate their clinical supervisors as well as perspectives on each method.

## Plain Language Summary

Genetic counseling programs in the U.S. and Canada evaluate their clinical supervisors using student feedback forms. All these forms are unique and have different goals. Peer‐review or self‐assessment methods are not widely used as they are difficult to put into practice, even though they are considered helpful. We suggest that clearer goals and well‐designed feedback forms could improve supervisor quality and strengthen students' clinical learning.

## 1 Introduction

Clinical supervision is an integral part of genetic counseling training programs. In the United States and Canada, students of genetic counseling programs (GCPs) must participate in a minimum of 50 cases under clinical supervision for the GCP to maintain accreditation with the Accreditation Council for Genetic Counseling (Accreditation Council for Genetic Counseling [ACGC] 2023, 12). This hands‐on experience allows students to interact with patients under the guidance of an experienced genetic counselor to develop essential clinical, interpersonal, and ethical competencies. With 64 ACGC accredited GCPs at the time of writing and over 500 new graduates entering the field in 2025, clinical supervisors play a critical role within the profession. Indeed, according to the NSGC's (2024) Professional Status Survey, 54% of genetic counselors working directly in patient care also serve as supervisors (National Society of Genetic Counselors [NSGC] 2024).

To maintain quality across training programs, the ACGC outlines expectations for clinical supervisors in its Standards of Accreditation.^1^ Supervisors must: “Establish fieldwork training goals specific to their setting; Define how students will be involved, supervised, and evaluated in client care and related activities; Observe, monitor, and evaluate student/client encounters; Provide an inclusive atmosphere conducive to student learning; Provide environments conducive to student learning; and Communicate with program leadership when situations of poor student performance arise; Incorporate principles of diversity, inclusion, equity, and justice into patient care and mentoring; Foster an inclusive environment where all individuals are valued and supported.” (ACGC 2023, 13). The ACGC also requires GCPs to regularly evaluate their clinical supervisors. However, the Standards do not specify the frequency, structure, or content of these evaluations. Additionally, clinical genetic counseling supervisors do not have standardized training or additional certification that would provide an external reference point for defining supervisory competencies, as exists in some other professions (American Association of Critical‐Care Nurses 2026; American Association for Marriage and Family Therapy 2023; Center for Credentialing and Education 2026). Programs must also allow students to provide feedback on each fieldwork site and anonymously evaluate their primary supervisors (ACGC 2023, 26–27). Here too, implementation is similarly ambiguous: student feedback may only be shared in aggregate and after a “sufficient” number of responses are collected to ensure anonymity (ACGC 2023, 26), providing challenges for programs that may only have one or two students for a given rotation in a year.

Attempting to evaluate genetic counseling supervisors is challenging, as clinical supervision is inherently complex compared to teaching in other contexts such as didactic lectures. Supervisors are responsible not only for clinical expertise but also for real‐time teaching, mentoring, and interpersonal guidance in emotionally sensitive settings. They must balance supervision with student autonomy, attend to emotional dynamics, navigate cultural and communication differences, and maintain appropriate professional boundaries (Caldwell et al. 2018; Wherley et al. 2015; Veach 2023). Unlike other health professions, genetic counseling supervisors often remain present throughout the entire patient session (ACGC 2023, 21), particularly early in training, creating nuanced demands for this one‐on‐one teaching dynamic (Landreville et al. 2019; O'Connell et al. 2025). The quality and impact of supervision involve many variables which are difficult to assess adequately. Furthermore, what constitutes adequate supervision may differ substantially from excellent supervision, but without more specific guidelines GCPs may be capturing these data inconsistently or inaccurately.

### 1.1 Methods of Evaluation

Given the variability of supervision evaluation needs and contexts, a range of evaluation methods have been employed in healthcare education more broadly, each with distinct advantages and limitations. These methods include student evaluation of teaching‐clinical^2^ (SET‐C), self‐assessment, peer evaluation, and management review. Student evaluations involve feedback from students at the end of a clinical rotation and, when well‐designed, can be valid and reliable, though they may be limited by bias, tool design, and supervisors' perceptions of accuracy (AlHaqwi et al. 2014; Copeland and Hewson 2000; Moore and Kuol 2005; Randall Armel 2025; Uttl et al. 2017). Self‐assessment encourages supervisors to reflect on their teaching, but is most effective when paired with other methods, as it may be undermined by inaccurate self‐perception or internal conflict (Eva et al. 2012; Fluit et al. 2013; Stalmeijer et al. 2010). Peer review entails observation and feedback by colleagues, offering benefits such as skill‐sharing and professional growth, though its success depends on institutional support, equitable peer relationships, and workplace dynamics (Bailey et al. 2014; Carroll and O'Loughlin 2014). In the professional experience of our team, many clinical supervisors are employed by clinical sites external to the university, making members of the GCP leadership responsible for fieldwork placements within their program—here called Fieldwork Directors (FDs)—unlikely to observe their supervisory performance directly. As a result, management reviews from an FD rely on existing documentation (SET‐Cs, peer input). Top‐down evaluations also tend to be sought when academic or career advancement within the institution is desired, rather than prompted by a need for assessment of clinical teaching skill. This may explain the limited presence of management review in the literature.

### 1.2 Study Overview and Aim

Despite the necessity of clinical supervision within GCPs as well as the ubiquity within the profession, there is limited information on the methods by which GCPs evaluate their clinical supervisors. Given the inherently complex nature of supervision and the accreditation requirement for evaluations, it is important to understand current evaluation practices to determine what tools are in use and if these tools are fit for purpose.

This study was part of a larger qualitative project aimed at understanding how genetic counseling programs evaluate clinical supervisors. We explored a range of evaluation approaches, their perceived strengths and challenges, and related practices such as feedback delivery, responses to negative evaluations, and recognition for high‐quality supervisors. Participants also shared evaluation tools, which we included in a document analysis. While the broader study collected data across multiple domains, this paper is focused specifically on answering the question of what methods genetic counseling programs are currently using to evaluate their clinical supervisors. To address this, we examined only data pertaining to the methods used by GCPs, the criteria guiding these evaluations, and analysis of the documents provided. To the best of our knowledge, this is the first document analysis and interview study on this topic within genetic counseling.

## 2 Methodology

We situated this qualitative study within a constructivist paradigm. This means that we viewed knowledge as co‐constructed between researchers and participants, shaped by context, perspective, and interaction rather than as a fixed, objective truth (Wainstein et al. 2023). This choice aligns with the research question, our desire to understand the range of practices, subjective perceptions, and contextual factors that influenced how evaluations occurred within GCPs, and our positionalities. This constructivist stance allowed space for diverse program structures, participant interpretations, and the meanings assigned to evaluation processes. We used the Big Q Qualitative Reporting Guidelines (BQQRG) as a framework for reporting our findings (Braun and Clarke 2025).

In recognition of the subjectivity inherent in constructivist qualitative research, and the essential role of the researchers in co‐creating knowledge, we offer the following positionality statement. The first author (SS) brought both personal and professional experiences that shape their perspective on clinical supervision in GCPs. As a white, nonbinary, socialized as female, queer individual, these intersecting identities influenced their interpretations of supervision and power dynamics. Conducting this research alongside clinical training aligned SS with the student perspective. Reflexive journaling was employed to bring awareness to this alignment and its potential impact on analysis. The research team also represented multiple perspectives, including program leadership, clinical supervision, and SA's current and JS's previous experience as ACGC board members, which contributed to richer interpretations and helped more holistically represent the data. These perspectives were reflexively incorporated into our analysis through dialogue among the research team.

### 2.1 Participants

Participants in this study were members of GCP leadership responsible for fieldwork placements within their program. While there were a range of titles for this position, for brevity we refer to these individuals as Fieldwork Directors (FDs) for the purpose of this study. FDs of genetic counseling programs across the United States and Canada were contacted via email through the Genetic Counselor Educators Association listserv with a request to fill out a demographic survey. A second email, serving as a reminder, was sent 3 weeks after the initial contact. Participants completed the demographic survey recording age of the program, location, size, and methods of supervision (in person, remote, or hybrid) provided by their program. Survey recruitment was conducted from October to December 2023. In November–December 2023 all survey respondents were invited via email to participate in an interview.

### 2.2 Data Generation

We developed a semi‐structured interview guide (Supporting Information S1) from a literature review and the professional experiences of the research team. SS conducted a pilot interview with a recently retired program director, which resulted in minor changes to wording for clarity. SS began all interviews with an introduction to the four common methods of supervisor evaluation as described in the Introduction, and invited participants to describe the methods their program utilizes as well as the content and the perceived effectiveness of their methods. They were also asked to provide their perspectives on any evaluation methods not utilized by their program. At the end of the interview, participants were given an opportunity to share copies of their tools for supervisor evaluation with SS for document analysis. Prior to the interview, each participant was given a copy of the interview guide for review. Each interview was audio‐recorded, transcribed by the transcription service Rev. AI, and edited by SS to de‐identify participants and correct any transcription errors.

### 2.3 Data Analysis

We used inductive content analysis (Vears and Gillam 2022) and interpretive description (Thompson Burdine et al. 2021) to generate findings. These methods were selected to allow for a broad range of responses given the narrow scope of this project and the gap within existing literature. Inductive content analysis first allowed us to generate descriptive categories based on the interviews. Interpretive description then provided a means through which we were able to translate our findings into a more accessible framework which GCPs could potentially apply to implement changes.

SS led the qualitative content analysis of all interview transcripts, following an inductive, question‐driven approach informed by Vears and Gillam (2022). In the initial organizational phase, SS collated responses into separate working documents according to the interview question addressed (e.g., all responses to “What methods does your program use to evaluate clinical supervisors?” were compiled together). This step functioned as a deductive structuring of the dataset by research question, enabling subsequent focused analysis. Within each document, SS conducted an inductive open‐coding process, systematically identifying and labeling distinct meaning units that captured the range of responses. Codes were iteratively compared, merged, and refined to develop subcategories that captured recurring patterns. These subcategories were then synthesized into higher‐order content categories that addressed the overarching research questions.

Sections requiring clarification were discussed with TW and SA in regular analytic meetings, ensuring consistent application of codes, enhancing the credibility of the findings, and incorporating researchers' positionalities and perspectives. The full team reviewed all coded material, refined category definitions, and collectively interpreted relationships between categories to construct a coherent representation of the data.

Once categories were identified through content analysis, interpretive description was used to create a conceptual model of how these codes were related to each other. This was achieved collaboratively through a series of discussion‐based team meetings.

A parallel document analysis was undertaken on the evaluation instruments that programs provided to students for assessing clinical supervisors. Each document was first catalogued for descriptive characteristics, including number of questions and the distribution of question types (scaled items, open‐ended written questions, and general comments). The content of each questionnaire item was then analyzed inductively using the same hierarchical approach as for transcripts: major codes captured the target of the question (e.g., supervisor‐focused vs. clinical rotation‐focused), and subcodes captured specific domains. The purpose of this phase was to characterize the content and structure of evaluation tools qualitatively rather than to generate numerical summaries or inferential statistics. Finally, the content of these student evaluation instruments was compared with corresponding interview transcript data to identify areas of overlap and divergence, providing a more comprehensive understanding of supervisory evaluation practices within and across programs.

### 2.4 Quality Practices

We used information power (Malterud et al. 2016) as a framework to consider the completeness of our analysis in responding to the aims of the study. Our participant group was limited to those who responded to the survey and subsequent invitation to an interview as well as the time constraints of a research study conducted in the context of a master's degree in genetic counseling. Nevertheless, we evaluated our study as having adequate information power based on the broad scope of the study; capturing variation in our participant group with respect to program age, class size, and location; not relying on existing theory; a high quality of interview dialogues; and a cross‐case analysis strategy with incorporation of the document analysis.

## 3 Results

### 3.1 Participant Characteristics

Twenty‐one participants completed the initial demographic survey. Given the lack of previous research, all survey respondents were offered an interview; sixteen participants agreed to participate, one declined, and four did not respond. Interviews lasted between 14 and 32 min with an average of 23 min.

Table 1 summarizes the demographic characteristics of the sixteen participating programs. Programs interviewed had a wide range of experience, ranging from less than 3 years to more than 20 years of operation. Class sizes ranged from 3 to more than 20 per graduating year, with an average of 10. Geographically, the programs were distributed across the United States and Canada with the majority in National Society of Genetic Counselors (NSGC) Region 4 (*n* = 4). All participants reported they used multiple methods of supervision such as in‐person supervision, remote supervision, or hybrid models. Ten of the sixteen programs provided blank copies of their SET‐C forms used to evaluate their clinical supervisors.

**TABLE 1:** Genetic counseling program demographics (*n* = 16).

Age of program (years)	
< 3	2
3–5	3
6–10	2
11–15	1
16–20	2
> 20	6
Current class size (*N*)	
3–5	3
6–10	8
11–15	3
> 16	2
Program location	
NSGC Region 1	3
NSGC Region 2	3
NSGC Region 3	1
NSGC Region 4	4
NSGC Region 5	3
NSGC Region 6	2

### 3.2 Review of Methods Used

SET‐Cs were the universal evaluation method, used by all 16 participating programs. The results provide a detailed analysis of this approach (Section 3.3), including what was being evaluated (Section 3.3.1), how evaluation data were collected from students (Section 3.3.2), and participants' perceptions of effectiveness (Section 3.3.3). The sections include both data from the analysis of the interviews and the document analysis of evaluation forms. The three remaining methods are then discussed in Section 3.4; self‐assessment (Section 3.4.1, used by three programs); peer evaluation (Section 3.4.2, used by one program); and management review (Section 3.4.3, not used by any programs), as well as their perceived strengths and weaknesses.

### 3.3 Student Evaluation of Supervisor

**FIGURE 1:**
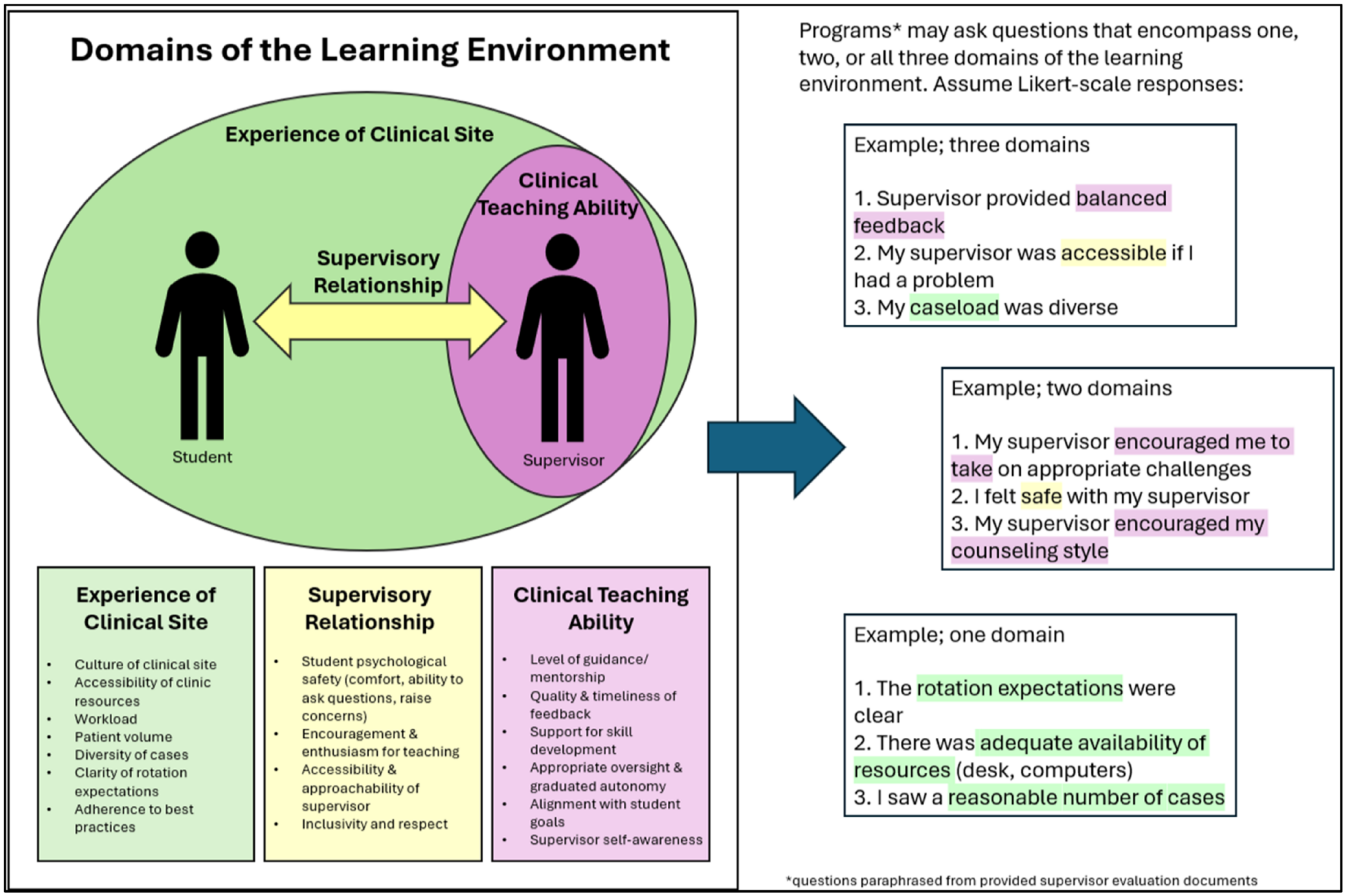
Conceptual Model of Student Evaluation of Teaching—Clinical in Genetic Counseling Programs. All questions in student evaluations regarded aspects of the learning environment, which are subdivided into three domains. The figure on the left represents the student providing the evaluation, and the figure on the right represents the supervisor being evaluated. The first domain, represented by the largest circle and green background represents the student's experience of the clinical site. The second domain is the supervisory relationship, represented by the yellow two‐way arrow between the student and supervisor. The final domain is clinical teaching ability, which is the purple circle encompassing only the supervisor.

Our conceptual model (Figure 1) reflects our understanding of the current use of SET‐Cs across the 16 programs included in the study. All questions in the student evaluations regarded aspects of the learning environment, which are subdivided into three domains. The first domain represents the student's experience of the clinical site. The second domain is the supervisory relationship. The final domain is clinical teaching ability. The components of the model are described in further detail below.

#### 3.3.1 Domains of the Learning Environment

Although our questions targeted the evaluation of clinical supervisors, participants reflected their SET‐Cs gathered data more broadly on evaluation, including questions regarding aspects of fieldwork sites that were not within the control of the supervisor. These data fit into a broader category termed here the learning environment (Figure 1). The learning environment consists of three distinct domains: the clinical site experience, supervisory relationship, and clinical teaching ability. As shown in the boxes on the right of Figure 1, participants reported that their programs typically evaluated at least two of these domains, some only targeted one domain, and others addressed aspects of all three.

From our document analysis, we saw that documents evaluating supervisors utilized a combination of questions in scaled and written format and spaces for general comments. The number of questions in these SET‐Cs ranged from 6 to 40 questions, with an average of 22. There was substantial variability in how questions were formatted and how response options were structured. Many forms employed Likert‐style scales, but the specific anchors and number of points were variable. Answers were available as a scale (e.g., Likert), Yes/No, or as open text boxes. Two SET‐Cs made space available for comment on scaled questions, while others made written comments mandatory if a respondent selected Disagree or Strongly Disagree. Questions with open text boxes were either phrased as specific questions (e.g., “Please indicate the supervisor's overall strengths”) or as spaces for general comments, either specified for the supervisor, the clinical rotation at large, or simply “additional comments.”

Many of the SET‐Cs in the document analysis explicitly asked about approachability or emotional safety (e.g., “I felt comfortable raising concerns with my supervisor”). In keeping with data reported during interviews, the document analysis demonstrated the majority of SET‐Cs contained both items pertaining to supervision and clinical site experience. Among these, supervisor‐related questions tended to make up the majority of the questions, although many approached a 50/50 split between the two. The majority of these SET‐Cs also included opportunities for general feedback on both the supervisor and a separate opportunity for general feedback on the site. Notably, however, one SET‐C asked questions exclusively about supervision. This SET‐C also contained an open space for comments regarding the rotation at large.

##### 3.3.1.1 Experience of Clinical Site

Represented by the green oval in Figure 1, questions about the clinical site experience evaluated aspects of the learning environment that were outside the supervisor's direct control, such as patient volume, site culture, and how prepared the student felt for a given rotation. This domain included the culture and safety climate of the site, the accessibility and adequacy of clinical resources, and logistical elements that affect day‐to‐day learning. Workload, patient volume, and the diversity of clinical cases determined both the pace and breadth of experiences available to students, while clear rotation expectations and structured orientation supported preparedness and engagement. This domain also covered elements of communication, such as the consistency of expectations across multiple supervisors and the clarity of instructions provided for the rotation.

Clinical site experience questions were present in most SET‐Cs documents reviewed, such as questions around case volume and diversity, site culture, and time provided to prepare for a session. These questions were either grouped under a clear subheading; others were interspersed throughout skill‐ or relationship‐based questions. One SET‐C utilized a separate survey for clinical site questions, and another SET‐C did not ask questions about the supervisor at all, although it did contain an open space for comments that could be related to anything regarding the rotation.

##### 3.3.1.2 Supervisory Relationship

The supervisory relationship, represented by the two‐way arrow in Figure 1, assesses the relational dynamic between the supervisor and the student. Central to this domain was the student's sense of psychological safety, such as the ability to ask questions or raise concerns without the fear of negative repercussions. This domain also explored the supervisor's approachability, accessibility, rapport, and perceived attitude toward supervision (e.g., if they were welcoming, enthusiastic). In this context, rapport referred not to likeability but rather the degree of harmony and effective communication between student and supervisor.

Did they feel comfortable? Did they feel safe? Did they feel supported? Did they also feel like they were able to develop that they were given the tools and the scaffolding and other supports to develop their tools? (P01)

##### 3.3.1.3 Clinical Teaching Ability

Clinical teaching ability, represented by the purple oval in Figure 1, evaluates the overall quality of the supervision provided. The clinical teaching ability domain is distinct from the supervisory relationship domain in that it points to aspects of providing supervision that may not be dependent on the relationship between the student and the supervisor or the perceived attitude of the supervisor toward supervision. For example, a student may have felt as though a supervisor was enthusiastic about clinical supervision (supervisory relationship domain), but provided feedback that was confusing (clinical teaching ability domain). Questions within this domain assessed the level of guidance and mentorship provided, the appropriateness of oversight, and the quality and timeliness of feedback offered to students. This participant explained, “I think we are trying to understand whether or not the supervisor is appropriate in the amount of oversight that they have over the case and whether or not they're jumping in too much, whether or not they're appropriately discussing prep and helping the student self‐reflect afterwards.” (P08).

Items also addressed how supervisors supported the development of clinical and professional skills, including graduated autonomy and the extent to which supervision aligned with the student's or the program's goals. Some evaluations included items asking the student to consider the supervisor's level of self‐awareness or reflection of their own teaching and clinical practice. One participant described for example, “…some of the [SET‐C] questions are asking if the supervisor… what some of their supervisory skills [are], do they have awareness of their own strengths and weaknesses as a supervisor?” (P02).

Most of the SET‐Cs in the document analysis asked questions regarding supervisor abilities. In several SET‐Cs, supervisor‐skill questions were grouped under a “supervisor” heading or a dedicated section, while others interspersed skill questions with relationship‐ or site‐specific questions. Most questions in this domain explore feedback, guidance, oversight, and support for skill development, while explicit items about supervisor self‐awareness were less frequent.

#### 3.3.2 Collection of Student Evaluations

When asked about how evaluation data were collected from students, participants reported using in‐person meetings between the student and their supervisor(s), anonymous SET‐Cs, or group discussions during end‐of‐year meetings with the entire cohort. Participants reported that additional insights into the student‐supervisor relationship could be obtained by reviewing student logbooks, either throughout the rotation or in bulk once a rotation had been concluded. Several participants mentioned they had one‐on‐one meetings with their students after each rotation, and described this as useful:

I also meet with them after every one of their rotations to go through it and then see if there's anything extra that they didn't feel comfortable addressing in their form. (P05)

Another participant mentioned that they can look at the supervisor's review of a student if the student rated a supervisor negatively. They described this can improve insight into the relationship dynamic:

I'm only getting one side [when viewing a student evaluation], but then we also get the supervisor's evaluation of the student. Sometimes you can see some correlations of both sides of that relationship where a student is expressing that a supervisor…was not particularly supportive or encouraging them to see unique things. But then you'll see in the student evaluation that they say…the student was not proactive about trying to do “fill in the blank”. So occasionally we can get a little bit of both sides of what that interaction was like. (P10)

#### 3.3.3 Efficacy of Student Evaluations

Participants' perceptions of the effectiveness of SET‐Cs were organized into three subgroups. The first group felt their tool for gathering the student perspective was effective and met the current needs of their program. The second group felt that it was an effective tool within certain limitations, such as the fact that students' perceptions changed over time while the evaluation only captured their opinion in a single moment. Some participants noted that individual personalities or specialty preferences (cancer, prenatal, etc.) influenced how students perceived a rotation, for which their evaluation tools may not account. For example, one participant described that “some students will always hate prenatal,” suggesting this could potentially lower ratings for any supervisor in a prenatal context. Several participants expressed that the effectiveness of a student evaluation was dependent on the student feeling safe and supported enough to be honest in their review, implying freedom from fear of backlash or retaliation from supervisors if a negative experience was reported. One participant described, “As an objective measure? I would say maybe not very well. The students are very careful in how they write things, and we as program directors really have to read between the lines. That said, on a practical level, students will contact us or drop hints when they are finding things not going great or they feel misunderstood or unhappy” (P06). In the third subgroup, participants felt their tool may not be effective, citing that students who fear retaliation may not be honest about a negative experience. This was exemplified by one participant who said, “I think it evaluates things quite well, but again, it requires honesty and vulnerability and people being comfortable taking risks. And that can only be done if supervisors created an appropriate environment in which to do that” (P15). Another participant felt that while their tool might differentiate poor supervisors from good ones, it may not distinguish between a good supervisor and an exceptional one.

To gain an understanding of if participants' evaluation tools were consistent with their experiences and perceptions of their SET‐Cs as they convey in their interviews, we compared the available SET‐Cs to their corresponding transcripts. Each program utilized a unique SET‐C; there were no duplicates shared across participating programs. We noted that many participants' verbal discussions of their evaluation processes/experiences aligned well with their corresponding SET‐C. For example, one participant felt they were gathering data on the supervisory relationship, the experience of receiving supervisor feedback, and functional site questions, which matched with the SET‐C questions related to appropriate expectations of the supervisor, time and energy, respect, and caseload;

We ask some criteria about the supervisor as an individual. Are they providing support? Are they open and encouraging? Are they welcoming? Do they provide balanced feedback? Those kinds of questions. And then we also ask some questions about this physical site in terms of is there a sufficient patient volume? Do you get a good variety? Do you have a space that is yours that you can work in? (P10)

The remaining comparisons represented a moderate amount of variability with respect to participants' views and their actual evaluation forms. For example, one participant described in their interview that the goal of their SET‐C was to assess student safety and how effective they felt supervisor feedback was. However, the questions in the SET‐C requested general or broad feedback without mentioning safety, feedback, or relational questions. Another participant emphasized the importance of understanding the relationship between student and supervisor in their interview, but the SET‐C document asked more practical questions around the performance of the supervisor and functionality of the clinical rotation.

### 3.4 Other Evaluation Methods

As described previously, other evaluation methods of self‐assessment, peer evaluation, and management review were used less frequently and are discussed here.

#### 3.4.1 Self‐Assessment

While many participants mentioned self‐reflection was a part of their program, only three participants described using a form of self‐assessment that was provided to all supervisors and incorporated into their overall performance review. Participants reported using institution‐specific tools, with none using established tools such as the Genetic Counseling Supervisor Self Efficacy Scale (GCSSES) (Spencer‐Hintze et al. 2023).

These three programs identified two purposes of self‐assessment. The first purpose was the professional development of the supervisor. For example, one institution required a triennial performance review of their supervisors which included a self‐assessment component. In this assessment, the supervisor outlines their strengths, weaknesses, and teaching goals for the upcoming three years. The institution then matches the supervisor with resources and support to help them achieve these teaching goals before their next performance review. The second purpose of self‐assessment was to gain insight into the dynamic between the supervisor and the student. In this case, the self‐assessments were performed annually and compared to the evaluations the supervisor received from students. These participants felt that a self‐assessment reflected a supervisor's *intent*, while perspectives from student evaluations demonstrated the supervisor's *impact* or effect on the student. This approach was described by a participant who expressed a desire to incorporate more formal self‐assessment into the review process for this purpose:

I really want their insight in how [supervisors] are thinking about what they're doing. I feel that in virtually every scenario of conflict I've encountered, it has been [sic]a absolute gap in communication [not a reflection of student skill]. The student is thinking and feeling one thing, the supervisor is thinking and feeling another thing. They both tell me and it's…just a full misunderstanding. (P14)

Additional FDs mentioned that self‐reflection or self‐assessments were being encouraged or performed in spaces like annual teaching retreats; however, these data were not shared with anyone and could not be incorporated into the overall assessment of the supervisor by the FD.

Programs using this form of assessment highlighted its benefits, including that it was logistically easy to send out to supervisors, and in conjunction with SET‐Cs created a dynamic assessment of supervisory performance. These sentiments were echoed by FDs who currently do not utilize a formal self‐assessment. While self‐assessment was generally regarded as positive, FDs discussed several limitations to this method. Notably, they felt self‐assessment relied on supervisor engagement and would therefore likely miss participants who need it the most. “That's the problem with self‐reflection. Right? The people that most need it are the ones least likely to do it.” (P07). Additionally, although it may be logistically straightforward to send out, enforcing their completion and retrieving completed forms from supervisors was cited as a challenge, especially for supervisors outside the participants' institutions. Programs not currently using self‐assessment expressed similar concerns around compliance/enforcement, and emphasized that any tool they added to their supervisors already heavy workload must be both effective and meaningful, particularly as supervisors receive no incentives for completing these assessments.

#### 3.4.2 Peer Evaluation

One FD reported using a form of peer evaluation as part of compliance with a state requirement to provide supervision over all aspects of work for any genetic counselor awaiting licensure. This included directly observing a genetic counselor as they engaged in supervision of a student. Several FDs mentioned using a form of peer mentorship, such as pairing a new supervisor with a more experienced supervisor for periodic discussion that did not involve direct observation, nor any evaluation. This practice evolved as a result of adjustments made during the start of the COVID‐19 pandemic, when a temporary measure to meet the increased demand for supervisors allowed genetic counselors with < 1 year of clinical experience to supervise students, provided they had a more experienced mentor. This is no longer practiced, but some programs found the pairing of a new supervisor with a more experienced mentor to be a valuable practice and continued it voluntarily. Some FDs reported that their institutions offered informal, voluntary, non‐evaluative peer supervision groups open to clinical supervisors across teaching disciplines. Others described peer learning in the form of annual or monthly meetings dedicated to peer‐to‐peer discussions, which also did not involve formal evaluation.

Programs that did not use peer evaluation highlighted that the format of supervision would be important to clarify, particularly over which aspects of supervision should be observed: the preparation before a session, the session itself, or the debrief afterward. Some felt only the debrief would be necessary, and some felt observing the entire session would be too much for the patient, or the student, or difficult logistically due to the limited space within clinic rooms. Others wondered if it would be helpful to observe a student with a mock patient, or observe a debrief with a mock student, to reduce stress for actual patients and students and focus on supervisor performance.

FDs recognized the potential benefits of incorporating peer evaluation, noting that it could create a more robust supervisor assessment, as the peer perspective is unique from that of the student and could capture aspects missed in a self‐assessment. Peer evaluation was considered particularly valuable for new supervisors, offering an opportunity for peer learning. Peer learning was regarded as very positive across multiple interviews.

Participants who did not utilize peer evaluation cited logistical challenges at the core of the decision. For example, participant 10 provided the following hypothetical:

The only hesitation is…do I even have the capacity? If we have 20 students…each one of them is doing three rotations, that's a minimum of 60 supervisors. Oftentimes their sites have more than one, so all of a sudden we're talking a hundred supervisors. Finding the manpower to be able to come in and directly provide supervision for a 100, 120, 130 people in one academic year? There's no way. Then how do I pick and choose which ones do you come in and see? Do I pick only those that have had something negative [in a student review]? (P10)

Other reported logistical challenges related to peer evaluation included barriers to scheduling, identifying peers, compliance, and justifying time away from clinic to managers. Others thought a peer evaluation would be ineffective because supervisors would act differently in front of a peer than they would otherwise authentically behave, or that supervisors would be reluctant to be observed after the constant observation they experienced in their own graduate training. Certain settings, particularly those with strict workplace hierarchies or existing tensions among colleagues, were considered unsuitable for peer evaluation, as these dynamics might worsen with peer‐based assessments. Lastly, there were concerns regarding the comfort and safety of both the student and the patient during a peer evaluation.

#### 3.4.3 Management Review

No programs described using a form of management review outside of supervisors who were applying for promotion within their institution. Some FDs mentioned they would write letters in support of promotion, but that this did not involve direct observation of clinic performance; rather looking at or pulling quotes from student evaluations. Student evaluations may also have been reviewed by leadership within programs in annual reviews, where supervisory performance was considered one of multiple dimensions of performance. However, several participants noted they were unfamiliar with the specific policies in external clinics affiliated with their program, suggesting that management review may be conducted at those sites independently.

Programs that did not utilize management reviews felt the observation of the supervisor by leadership could be a helpful tool when assessing candidates for promotion. Others felt it would be challenging logistically, particularly when trying to coordinate with clinics outside their institution, where responsibility and jurisdiction over issues of quality or performance may become blurred.

## 4 Discussion

This study is the first to explore the phenomenon of how genetic counseling programs evaluate clinical supervisors. Our findings illuminate patterns, gaps, and challenges in current evaluation practices. Our findings clearly demonstrate that programs differ greatly regarding the perceived purposes and methodologies of these evaluations. While student evaluations were widely used, their content, structure, and purpose varied significantly across programs. In contrast, self‐assessment and peer evaluation were rarely implemented, despite their perceived potential to enrich feedback and support supervisor development.

Our conceptual model and analysis of other evaluation methods reflect the variability in supervisor evaluation models and their lack of consistency across programs, likely due to limited guidelines and available resources. While it is unclear whether this variability is inherently problematic, it suggests a lack of consensus around what constitutes effective supervision and how it should be evaluated. Without clearer guidance from accrediting bodies, available frameworks, or explicit program‐level clarity regarding what is being measured and for what purpose, evaluations risk failing to capture meaningful indicators of supervisory effectiveness. To ensure high‐quality training and equitable learning environments, genetic counseling programs must critically examine their evaluation practices and invest in tools that are purposeful, practical, and aligned with the complexity of supervision in this field. These results are crucial for our understanding of effective clinical supervision in genetic counseling programs. By understanding the landscape portrayed in our conceptual model, programs can identify areas for improvement and implement changes that are contextually appropriate and actionable, such as clarifying evaluation objectives, adopting complementary assessment methods, or developing tools that better support supervisor development.

### 4.1 Ubiquity of Student Evaluation of Supervisors

All interviewed programs utilized SET‐C, a method widely used across healthcare disciplines, including medicine, nursing, and allied health professions (Bartlett et al. 2020). The current guidelines for student evaluations have led each GCP to develop distinct approaches to this method. When considered collectively, student evaluation questions reflected a broad range of learning environment domains and can contribute to a nuanced understanding of the supervisory dynamic. However, individually some programs focus narrowly on a single domain, which raises questions about the appropriateness of these instruments for evaluating overall supervisory effectiveness. As a result of this variability evaluations may be ambiguous, making it difficult for supervisors to interpret feedback, address areas for improvement, or ensure equitable learning experiences for students across programs.

One solution could be for the Genetic Counselor Educators Association or the ACGC to establish a shared understanding of the objectives of student evaluations, clarifying whether the goal is to assess the student experience, support supervisor development, or both. Supervisory expectations could involve the current literature on supervisory competencies or competencies from similar professions (Bartlett et al. 2020; Eubanks Higgins et al. 2013; Wherley et al. 2015). It is crucial to recognize the limitation of SET‐C to prevent misuse; students, given their stage of professional development, may not be able to directly evaluate a supervisor's clinical judgment, but can provide valuable feedback regarding the social and emotional aspects of the learning environment. Complementary approaches, such as supervisor self‐assessment, peer review, or multisource feedback, could address gaps students are unable to assess, such as clinical judgment or teaching strategy. Although this was not the focus of our document analysis, we acknowledge the value of applying validated survey design principles (e.g., avoiding double‐barreled or leading questions) which could enhance the accuracy, interpretability, and actionability of feedback. Together, these strategies can help programs ensure that evaluations provide meaningful, actionable insights that support supervisor growth and improve the quality of clinical training.

Efforts to improve evaluation tools must also account for structural factors that influence supervisory performance. Supervisor evaluation is complicated by the lack of structured training models for new and current genetic counseling supervisors. Although some form of training is required, expectations are vaguely defined, and no formalized or standardized supervisor training programs currently exist (ACGC 2023, 12). Without clear behavioral expectations established through training, it is difficult to determine which aspects of supervision should be evaluated. Compounding this issue is the increasing prevalence of burnout in genetic counseling and other healthcare professions (Caleshu et al. 2022). Supervision adds to an already demanding workload, and many supervisors receive no formal compensation or incentives, such as promotions, for this additional responsibility. Additionally, we can extrapolate that non‐institutional supervisors who work with students across multiple GCPs and across geographic locations (in virtual settings) must therefore manage differing supervisory expectations and models of feedback; unifying these goals and tools may help reduce associated burnout. These structural and systemic factors may affect supervisory performance and must be considered in the development of evaluation tools.

In classroom settings, student evaluations of teaching (SET) have demonstrated validity and reliability when properly structured, and students may feel more empowered to provide feedback in safe, confidential environments, especially when validated tools are used (AlHaqwi et al. 2014; Copeland and Hewson 2000; Fluit et al. 2013). However, SET alone may not be sufficient to drive change, as it can be affected by design limitations, potential bias against supervisors with marginalized identities, and supervisors' perceptions of feedback accuracy (Choshi et al. 2023; Esarey and Valdes 2020; Stalmeijer et al. 2010; van Lierop et al. 2018; Uttl et al. 2017). While consistent use of SET can help assess supervisory competencies and measure change over time, attributes such as empathy or enthusiasm remain difficult to quantify. Comparisons to other allied health professions are limited because genetic counseling supervision includes unique elements, such as high rates of direct observation and co‐counseling. Fields with partially similar supervision structures, including speech‐language pathology, psychology, social work, and occupational therapy, also use SET‐C and, in some cases, validated tools such as the Supervisory Working Alliance Inventory–Trainee Form, Manchester Clinical Supervision Scale, and the Clinical Supervision Evaluation Questionnaire (Horton et al. 2008; Sabella et al. 2020; Winstanley and White 2011). Across healthcare programs, matching specific competencies with appropriate evaluation methods remains challenging, and evidence linking supervisor competencies to student outcomes is limited. The wide range of tools used by genetic counseling programs is not surprising given limited guidelines. But further research is needed to determine whether this variability is beneficial, allowing room for the local needs of a program to be addressed, or if it undermines consistency and the establishment of universal standards of supervisory quality.

#### 4.1.1 Document Analysis

Document analysis revealed similar variability in the design and structure of SET‐Cs. SET‐Cs often included multiple domains, reflecting both supervisory performance and broader aspects of the learning environment. Additionally, integrating supervisor reviews with overall clinical site reviews is a possible confounding factor in supervisor assessment (see Section 4.2). These findings highlight the diversity of current practices and underscore the importance of considering field‐wide approaches to support programs in developing more consistent and actionable evaluation instruments. However, given the resource constraints already facing the field, this additional work may not be feasible at the individual program level. This underscores the need for broader field‐wide reflection and coordinated research efforts to develop validated and reliable evaluation instruments.

### 4.2 Conflation of SET‐C With Evaluation of the Learning Environment

Our analysis indicates that SET‐Cs in the participating GCPs typically included questions addressing both supervisor performance and broader aspects of the learning environment, including elements outside the supervisor's control such as case diversity, rotation logistics, or institutional culture. When student evaluations cover both the supervisor and overall environment, feedback specific to the supervisor may become conflated with experiences of the site, potentially reducing the actionability of the feedback or misrepresenting the supervisor's performance.

Currently, there are no established best practices for supervision evaluation in genetic counseling, though quality survey design includes defining operational terms and clarifying the purpose of each item to align questions with specific outcomes (Ziniel et al. 2019). Further research is needed to understand whether conflating supervisor assessment with broader learning environment factors affects feedback, and whether strategies such as using subheadings or separate instruments could mitigate potential confusion. Collecting data from multiple sources, including supervisor self‐reflection or peer review, may help reduce the influence of site‐level factors on supervisor evaluation, similar to multisource feedback approaches in other allied health professions (Pelgrim et al. 2011). Additional research is needed to identify the most effective methods for distinguishing supervisor performance from environmental variables while maintaining actionable, meaningful feedback for supervisor development.

### 4.3 Use of Non‐Student Evaluation Methods Less Known

In contrast to student evaluations, methods such as self‐assessment, peer evaluation, and management review were less commonly reported across programs. Each came with their own perceived strengths and weaknesses, as well as barriers to implementation.

#### 4.3.1 Self‐Assessment

Several programs described informal self‐reflection among supervisors, though few had formalized processes integrated into performance reviews. While participants generally viewed self‐assessment positively, noting its potential to support professional development and provide insight into the supervisor‐student dynamic, concerns were raised about engagement, compliance, and added workload. The literature indicates that self‐assessment alone is limited by tendencies toward self‐criticism, overestimation of ability, and the influence of social desirability, but when paired with complementary feedback such as student or peer evaluations, it can enhance professional growth (Eva et al. 2012; Finn et al. 2011; Fluit et al. 2013; Stalmeijer et al. 2010). There are several validated tools for assessing supervisory skill, including the recently developed GCSSES and the Psychotherapy Supervisory Development Scale (Spencer‐Hintze et al. 2023; Watkins Jr. et al. 1995). Although each program was asked if they used either of those tools, none did. However, the GCSSES was a relatively new tool at the time of the interviews. For integration, other health care professions have suggested embedding reflective practice within existing evaluation structures, such as annual reviews or multisource feedback cycles, framing it as a formative, developmental activity supported by faculty training, rather than a stand‐alone administrative task (Bartlett et al. 2020). As with the SET‐Cs, any self‐reflection tool for genetic counselors would require clear guidance on purpose and intended outcomes, as well as research to determine which needs can be effectively addressed through self‐assessment or reflective practice.

#### 4.3.2 Peer Evaluation

Interview participants frequently referenced peer evaluation and peer learning, at times interchangeably. Peer evaluation typically involves one supervisor formally observing another for the purpose of assessing performance (Doroh et al. 2023; White et al. 2025), whereas peer learning refers to collaborative, non‐evaluative activities like mutual observation followed by reflective discussion (Saab et al. 2021). Participants generally viewed peer learning as more favorable and flexible to implement, while peer evaluation, although also perceived as positive, was likely too logistically challenging to institute and may not be well‐received by supervisors. Studies show that peer evaluation can provide the opportunity to acquire new teaching techniques from colleagues, foster professional development, and strengthen workplace relationships (Bailey et al. 2014; Finn et al. 2011), but is dependent on institutional support, workload feasibility, and peer dynamics (Carroll and O'Loughlin 2014; Murphy Tighe and Bradshaw 2013; Saab et al. 2021). Similar challenges have been described in other allied health disciplines, where peer observation is recommended as part of a broader, portfolio‐based multisource framework rather than routine practice (Bartlett et al. 2020). In this context, peer review and peer learning serve complementary functions: the former providing structured feedback for growth, and the latter fostering continuous reflective dialogue and shared teaching strategies. Others suggest that peer observation should not be used for evaluative purposes, but rather as a tool for self‐improvement (Berk et al. 2004). More research is required to assess the needs and capacity for peer evaluation within genetic counseling.

#### 4.3.3 Management Review

A few programs mentioned administrative or “top‐down” evaluations, though these were not well characterized and often relied on aggregate student or peer reports rather than direct observation. This approach aligns with evidence from other healthcare disciplines, where administrative reviews are typically tied to performance management, hiring, or promotion rather than formative skill development (Hewko and Cummings 2016; Krijgsheld et al. 2022). Some participants addressed the challenges associated with being unable to compensate supervisors financially for their work with students, and the limited oversight possible for supervisors not associated with the university. While some had enough supervisors to feel empowered to decline further association with a supervisor who is not a good fit, others described feeling pressure to maintain positive relationships due to the limited availability of supervisors. Theoretically, FDs in the latter position may be disinclined to investigate negative reviews for fear of losing that resource. Further exploration would be required to understand how such evaluations could be aligned with supervisor development goals while maintaining fairness and transparency.

Together, these findings indicate that non‐student evaluation methods are recognized but underutilized in genetic counseling education. Broader research across ACGC‐accredited programs could help understand how to best adapt existing multisource and peer‐based models to the unique supervisory context of genetic counseling.

### 4.4 Practical Implications

To strengthen the evaluation of clinical supervisors, genetic counseling programs can begin by clearly defining the purpose of supervisor evaluations within their institution (whether to assess the student experience, support supervisor development, etc.) to move toward alignment between tools and intended outcomes. Our analysis suggests utilizing structured and, when available, validated evaluation instruments would improve reliability and comparability of feedback. GCPs can also explore incorporating complementary methods such as structured self‐reflection and peer review, embedding these into existing review cycles or professional development activities to minimize additional workload and support supervisor growth. Together, these steps can help programs generate meaningful, actionable evaluations that promote high‐quality supervision and equitable learning experiences for students.

### 4.5 Strengths and Limitations

This is the first study to systematically explore the methods used by genetic counseling programs to evaluate their clinical supervisors, adding important and novel findings to the literature about clinical supervision in genetic counseling. The ability to compare interview data with SET‐C documentation from many participants allowed us to deepen our analysis of how students were evaluating genetic counseling supervisors. These data resulted in the identification of actionable areas for improvement, both on a program‐ and accreditation‐level.

The study was limited in that it relied predominantly on self‐reporting of FD and voluntary document submissions, which may reflect a self‐selecting group predisposed to engagement. It is possible that participation bias influenced our analysis. FDs who participated in interviews may have had a greater interest in supervision with, perhaps, more consistent attention to evaluation of clinical supervisors. The perspectives of FDs who had not considered these topics as thoroughly may be less likely to be included here. Some FDs were also clinical supervisors, which could have created a difference of opinion not accounted for in our analysis. While SET‐C forms were reviewed, technical validation of the instruments was not performed, limiting any conclusions regarding quality.

### 4.6 Future Research

While this study identified current evaluation practices, gaps remain in understanding how these tools are experienced and applied. Future studies should include the perspectives of both clinical supervisors and students to better understand how evaluation tools are experienced, interpreted, and applied. Student perceptions of anonymity, emotional safety, and the usefulness of feedback processes may clarify barriers to honest reporting, while supervisors' experiences receiving and responding to feedback, particularly in the absence of training or incentives, warrant closer examination. Additionally, factors such as resource availability, supervisor burnout, and program structure could be explored as influences on evaluation practices.

Finally, given the positive perceptions of informal self‐reflection and peer learning, future work should explore how these practices might be intentionally structured and supported. Future studies could help determine whether formative, nonpunitive feedback models foster greater supervisor engagement and development than other approaches. Together, these areas of investigations could help build more effective, equitable, and sustainable evaluation frameworks.

### 4.7 Conclusion

This study underscores the need for greater alignment and clarity in how clinical supervisors are evaluated across genetic counseling programs. Although flexible accreditation standards allow programs to tailor evaluation tools to their contexts, they also contribute to inconsistency in what is measured, how feedback is collected, and how results are applied. Clarifying the underlying purpose of supervisor evaluations and establishing shared frameworks and validated tools are essential next steps to ensure evaluations are actionable and aligned with their intended goals.

## Author Contributions

**Sydney Schulz:** conceptualization, data curation, formal analysis, funding acquisition, investigation, methodology, project administration, validation, visualization, writing – original draft, writing – review and editing. **Jenna Scott:** conceptualization, formal analysis, funding acquisition, methodology, project administration, supervision, writing – review and editing. **Tasha Wainstein:** formal analysis, methodology, supervision, validation, writing – review and editing. **Tracey Oh:** conceptualization, formal analysis, funding acquisition, methodology, project administration, supervision, writing – review and editing. **Susan Randall Armel:** formal analysis, methodology, supervision, validation, writing – review and editing.

## Funding

This study was funded by the University of British Columbia Department of Medical Genetics.

## Disclosure

*AI Statement*: Artificial Intelligence Generated Content (AIGC) tools were not used in the preparation of this manuscript.

## Ethics Statement

This research study was approved by the Children's and Women's Research Ethics Board (REB) of the University of British Columbia (H23‐01753‐A002). Informed consent was obtained from all participants included in the study.

## Conflicts of Interest

The authors declare no conflicts of interest. Tasha Wainstein is the Deputy Editor of the *Journal of Genetic Counseling* and a co‐author of this article. She was excluded from editorial decision‐making related to the acceptance of this article for publication in the *Journal*.

## Supporting information


**Data S1:** Supporting Information.

## Data Availability

The data that support the findings of this study are available on request from the corresponding author. The data are not publicly available due to privacy or ethical restrictions.

## Endnotes

^1^
This study reflects the 2023 Standards of Accreditation, in use at the time interviews were conducted.

^2^
Previous literature describing student evaluation of educators uses the term Student Evaluation of Teaching and is typically applied to a classroom‐based education. Here we use Student Evaluation of Teaching—Clinical to differentiate our research as focused on the functionally different, often 1:1 dynamic required for clinical supervision.
