# Supplementary material for: Current Practices for Evaluating Clinical Supervisors in North American Genetic Counseling Programs
Source: J Genet Couns. 2026 Aug 23;35(4):e70280. doi: 10.1002/jgc4.70280 (PMC13500935; doi:10.1002/jgc4.70280)
Supplement: Supplementary file 1 [file JGC4-35-0-s001.docx]

**Supplementary Material**

Title: Current Practices for Evaluating Clinical Supervisors in North American Genetic Counseling Programs

**Sydney Schulz**, MSc, CGC (they/them), Department of Medical Genetics, Faculty of Medicine, University of British Columbia, Vancouver, BC, Canada, ([schulzs617@gmail.com](mailto:schulzs617@gmail.com))

ORCID: 0009-0006-2696-0853

**Tasha Wainstein**, PhD, MSc (Med), CCGC (she/her), Departments of Pediatrics and Medical Genetics, Faculty of Medicine, University of British Columbia, Vancouver, BC, Canada. BC Children’s Hospital Research Institute, Vancouver, BC, Canada ([tasha.wainstein@bcchr.ca](mailto:tasha.wainstein@bcchr.ca)),

ORCID: 0000-0002-4890-9657

**Susan Randall Armel**, MSc, MS, MScCH, CGC, (Department of Molecular Genetics, University of Toronto), Toronto, ON, Canada; Bhalwani Familial Cancer Clinic, Princess Margaret Cancer Centre, Toronto, ON, Canada ([susan.randall@uhn.ca](mailto:susan.randall@uhn.ca))

ORCID: 0000-0001-5016-3148

**Jenna Scott**, MSc, (she/her-), Genetic Counselling Program Co-Director, University of British Columbia, Vancouver, BC, Canada, (jscott3@cw.bc.ca)

ORCID: 0000-0003-1631-332X

**Tracey Oh**, MS, CGC, (she/her), Department of Medical Genetics, Faculty of Medicine, University of British Columbia, Vancouver, BC, Canada, (toh@cw.bc.ca)

ORCID: [0009-0002-6809-5803](https://orcid.org/0009-0002-6809-5803)

**Interview Guide**

 

1. Keeping these methods in mind, can you describe how you evaluate your clinical supervisors?
2. Does the evaluation method change if the mode of supervision is different? For example, you have one method for in-person rotations and another for hybrid rotations.
3. Questions for each modality used:
   a. How long have you used this modality?
   b. What are you trying to evaluate with this method? What criteria are you using to evaluate their skills?
      i. Prompts: “Some programs are investigating how the student felt, or assess supervisory competency (e.g. the Higgens et al supervisory competencies)”
   c. How well do you think this measures [what they’re trying to evaluate]?
   d. What do you feel are the strengths or advantages of this method?
   e. What would you say are the disadvantages?
   f. How frequently do you provide feedback to a supervisor specifically? [not site-specific]
      i. Do you feel like this is often enough or would you prefer to do it more often?
      ii. Is the feedback edited or modified before given to the supervisor?
   g. Do you have a protocol for feedback that is negative? (Assuming it’s not a policy violation or something actively concerning)
      i. Do you wait for it to be consistent?
   h. Is there any advantage or incentive to feedback that is consistently positive?
   i. If they use self-assessment: Do you use established tools like the genetic counseling supervisory self-efficacy scale or Psychotherapy Supervisor Development Scale?
4. What do you think of the other methods of supervision?
   a. Would you consider implementing one of the other methods?
   b. What barriers do you see to using this method?
   c. Do you see advantages?

General Closing:

1. Would you be open to sharing your tools with us for a document analysis? We would not be explicitly performing your program.
2. Would you like to be notified if we publish our results?

**Figure 1 Color Palette**

The color palette for the visual aid in Figure 1 was designed to be accessible to individuals with colorblindness. The selected colors are distinguishable for viewers with protanopia, deuteranopia, or tritanopia, and all data points are further differentiated through distinct shapes and labels. This image was created via davidmathlogic.com/colorblind/
